# A review of the management of posterior communicating artery aneurysms in the modern era

**DOI:** 10.4103/2152-7806.74147

**Published:** 2010-12-22

**Authors:** Kiarash Golshani, Andrew Ferrell, Ali Zomorodi, Tony P. Smith, Gavin W. Britz

**Affiliations:** Department of Radiology, Division of Vascular and Interventional Radiology, Duke University Medical Center, Durham North Carolina, USA; 1 Department of Surgery, Division of Neurological Surgery, Duke University Medical Center, Durham North Carolina, USA

**Keywords:** Cerebral aneurysm, clipping, coiling, posterior communicating artery

## Abstract

**Background:**

Technical advancements have significantly improved surgical and endovascular treatment of cerebral aneurysms. In this paper, we review the literature with regard to treatment of one of the most common intra-cranial aneurysms encountered by neurosurgeons and interventional radiologists.

**Conclusions:**

Anterior clinoidectomy, temporary clipping, adenosine-induced cardiac arrest, and intraoperative angiography are useful adjuncts during surgical clipping of these aneurysms. Coil embolization is also an effective treatment alternative particularly in the elderly population. However, coiled posterior communicating artery aneurysms have a particularly high risk of recurrence and must be followed closely. Posterior communicating artery aneurysms with an elongated fundus, true posterior communicating artery aneurysms, and aneurysms associated with a fetal posterior communicating artery may have better outcome with surgical clipping in terms of completeness of occlusion and preservation of the posterior communicating artery. However, as endovascular technology improves, endovascular treatment of posterior communicating artery aneurysms may become equivalent or preferable in the near future. One in five patients with a posterior communicating artery aneurysm present with occulomotor nerve palsy with or without subarachnoid hemorrhage. Factors associated with a higher likelihood of recovery include time to treatment, partial third nerve deficit, and presence of subarachnoid hemorrhage. Both surgical and endovascular therapy offer a reasonable chance of recovery. Based on level 2 evidence, clipping appears to offer a higher chance of occulomotor nerve palsy recovery; however, coiling will remain as an option particularly in elderly patients or patients with significant comorbidity.

## INTRODUCTION

Posterior communicating artery (PCOM) aneurysms are one of the most common aneurysms encountered by neurosurgeons and neurointerventional radiologists and are the second most common aneurysms overall (25% of all aneurysms) representing 50% of all internal carotid artery (ICA) aneurysms.[40] Not only these aneurysms can present with a typical subarachnoid hemorrhage, but also they can present with an isolated occulomotor nerve palsy (OMNP) or a non-traumatic subdural hematoma (SDH). In addition, because of the variation in the anatomy of this aneurysm and its parent arterie(s), these aneurysms can be among the easiest and the most difficult to treat either surgically or endovascularly. The purpose of this paper is to review the unique qualities of this aneurysm with respect to anatomy, natural history, and treatment.

## ANATOMY

There are considerable variations in the anatomy of the PCOM complex, which have both surgical and endovascular implications. The size of the PCOM artery ranges from a small artery often not visualized on angiography, to a large artery nearly the size of the posterior cerebral artery (PCA). A fetal PCOM variant is defined as a PCOM artery, which has the same caliber as the P2 segment of the PCA and is associated with an atrophic P1 segment. Because fetal PCOM arteries are the primary supply to the PCA, care must be taken not to compromise flow to this artery during clipping or coiling of PCOM aneurysms. The incidence of the fetal PCOM variant is 4-29% of patients and bilateral fetal PCOM variants occur in 1-9% of patients.[6,21,24,46,52] There is also considerable variation in the extent of involvement of the PCOM artery by the neck of the aneurysm. The aneurysm may be similar to sidewall ICA aneurysm in which the PCOM origin is just distal to the neck of the aneurysm. However, the most commonly encountered variation occurs when the neck of the aneurysm partially incorporates the PCOM artery. Rarely, the neck of the aneurysm may originate solely off of the PCOM artery itself. This variant is referred to as a “true PCOM aneurysm” and has a reported incidence of 0.1-2.8% of all aneurysms (4.6-13% of PCOM aneurysms).[19,29,38,51] These aneurysms are often associated with large or fetal PCOM arteries and treatment of these aneurysms require significant diligence in order to maintain flow in the PCOM artery.[19]

The direction of the dome also has significant surgical implications. A classification system has been described by Yasargil.[59] When the fundus points anteriolaterally, the origin of the PCOM artery may be hidden by the aneurysm. In addition, the aneurysm may be adherent to the clinoid process requiring careful dissection so that the fundus may be mobilized and the origin of the PCOM is visualized during application of a clip. A superiolateral fundus is occasionally encountered in which the dome points into the sphenoid ridge. Hemorrhage of these aneurysms may cause a subdural hematoma. In contrast, a posterolateral superior fundus points into the temporal lobe and is often associated with an intraparenchymal hemorrhage and/or hemorrhage into the temporal horn of the ventricle. A posteriolateral inferior fundus often penetrates the Liliquist membrane pointing into the interpeduncular fossa. These aneurysms are also associated with OMNP. A posteriomedial inferior is rarely encountered. These aneurysms are often sidewall aneurysms of the ICA.

## NATURAL HISTORY

Although patients with a PCOM aneurysm typically present with subarachnoid hemorrhage (SAH), they can also present with SDH or an isolated OMNP. Although the pattern of SAH is often typical involving the basal cisterns, PCOM aneurysms may present with isolated hemorrhage in the temporal lobe, temporal horn of the lateral ventricle or sylvian fissure (mimicking a middle cerebral artery aneurysm). In addition, 1.3-7.9% of aneurysms may present with an SDH, and a PCOM aneurysm responsible for the hemorrhage is 43% of cases (2/3^rd^ of convexity SDH).[14,34,39,45]

The risk of rupture of cerebral aneurysm was recently evaluated by the ISUIA.[56] In this report, the risk of rupture of posterior circulation aneurysms [<7 mm (2.5%), 7-12 mm (14.5%), 13-24 mm (18.4%), >25 mm (50%)] was found to be higher than that of anterior circulation aneurysms [<7 mm (0%), 7-12 mm (2.6%), 13-24 mm (14.5%), >25 mm (40%)]. PCOM aneurysms were grouped with the posterior circulation aneurysms with respect to risk of rupture. However, this study had significant limitations. A selection bias existed in which only patients chosen for conservative management by their treating physician were included. This will tend to exclude aneurysm with irregular shapes and include aneurysms in difficult locations (such as the posterior fossa). In addition, there is crossover of patients into the treatment group, and premature death of patients for reasons unrelated to the aneurysm. The increased risk of rupture for PCOM aneurysms is due to two patients who had rupture of their PCOM aneurysm during the follow up. Clark *et al*, completed a meta-analysis of patients with unruptured aneurysms and found that when the ISUIA cohort is excluded, the rate of rupture for PCOM aneurysm (0.46% per year) mimics that of anterior circulation aneurysms (0.49% per year) rather than posterior circulation aneurysms (1.9% per year).[10] In addition, the ISUIA study gives the impression that small aneurysms have a minimal risk of rupture. However, Forget *et al*, reviewed the sizes of ruptured aneurysms and found that 85.6% of these aneurysms were less than 10 mm. The prevalence of small ruptured PCOM aneurysms was particularly high with 87.5% of aneurysms measuring less than 10 mm in diameter and 40% measuring less than 5 mm.[12] He *et al*, compared the prevalence of rupture between true PCOM aneurysms and junctional PCOM aneurysms (where the neck of the aneurysm involves both the ICA and PCOM artery).[19] Although no difference was found between the two groups, the average size of true PCOM aneurysms was significantly smaller than that of junctional PCOM aneurysms. Based on the association of aneurysm size and rate of rupture, the author extrapolated that true PCOM aneurysm may have a higher risk of rupture than junctional PCOM aneurysms; however, there was no significant difference in the prevalence of rupture when size-matched cohort of junctional PCOM aneurysms were compared to true PCOM aneurysms. In conclusion, the rate of rupture of PCOM aneurysms is similar to other anterior circulation aneurysms, and a small size is not necessarily an indication of a low rupture risk. The rate of rupture is artificially low in the ISUIA study due to the many biases of the study. The actual rate of rupture is probably closer to that reported in a Finnish article.[25,26] Although many of the patients in this study harbored multiple aneurysms and the diversity of the population in this study is limited, this study lacks bias in terms of aneurysms that are treated and followed. The reported risk of rupture is 1.4% per year.[25,26]

Jane *et al*, evaluated the risk of rehemorrhage in ruptured ACOM and PCOM aneurysm, finding a 50% risk of rerupture within the initial six months followed by 3.5%/year thereafter.[22] Interestingly, the author found an association between hypertension and rebleeding in the PCOM cohort. The reason for this association is unclear but one can hypothesize that blood pressure is more easily transmitted to the dome of aneurysms of primary vessels (I.e. ICA) than secondary vessels (I.e. anterior cerebral artery).

### Surgical Clipping of PCOM Aneurysms

PCOM aneurysms can be one of the easiest or one of the most difficult aneurysms to treat surgically. The PCOM artery is one of the first branches visualized during dissection of the carotid cistern and the dome of the aneurysm is typically directed away from approach trajectory. Wide dissection of the sylvian fissure is typically not necessary for successful clipping of these aneurysms. In fact, retraction of the temporal lobe is often avoided (particularly when the fundus points laterally) until the surgeon partially exposes the neck of the aneurysm. Anterior cliniodectomy is rarely required for clipping of PCOM aneurysms. Park *et al*, found that only 6 out of 96 patients with PCOM aneurysms required clinoidectomy during surgical treatment.[42] When looking for predictors of possible clinoidectomy, he found three anatomical factors. The distance between the tip of the anterior clinoid process and the proximal neck of the aneurysm (measured using CTA) was significantly smaller in the clinoidectomy group (4.4 ± 0.7 mm *vs* 7.2 ± 1.4mm). From this data, one can extrapolate that if the distance is greater than 5.8 mm, anterior clinoidectomy is rarely required, and when the distance is less than 4.4 mm, anterior clinoidectomy is frequently required for successful clipping. The other anatomical measurements are somewhat difficult to measure of digital subtraction angiography. The data indicates that the communicating segment of the ICA take a more lateral course in the clinoidectomy group.

Once the neck of the aneurysm is adequately exposed, the surgeon must pay significant attention to preservation of the PCOM artery, PCOM perforators and the anterior choroidal artery without significant manipulation of the fundus. Leipzig *et al*, reviewed a large series of aneurysm clipping looking for risk factors of intra-operative rupture.[32] PCOM aneurysms had the second highest rate of intra-operative rupture (second only to ACOM aneurysms) amongst anterior circulation aneurysms (9.3%). Risk factors for intra-operative rupture included an immediate history of subarachnoid hemorrhage as well as lack of temporary clipping. PCOM aneurysms in particular had a significantly higher incidence of intraoperative rupture when no temporary clip was used during clipping of the aneurysm (11.6% *vs*. 0%). One reason for this finding is that the fundus of the PCOM aneurysm is typically in line with the communicating segment of the ICA allowing a significant portion of the intra-arterial pressure to be applied to the dome of the aneurysm. The communicating segment of the ICA begins just proximal to the origin of the PCOM artery and ends in the ICA terminus. This also explains why ligation of the cervical ICA is protective of rehemorrhage for PCOM aneurysms.[57] As an alternative to temporary clipping, adenosine-induced cardiac arrest may also be used to temporarily reduce the pressure within the aneurysm during application of a clip.[17,43]

This technique is particularly useful for PCOM aneurysm because of the number of vessels that can provide high pressure inflow into the aneurysm. For instance, temporary proximal to an MCA aneurysm will significantly reduce the pressure within the fundus. Similarly, temporary clipping of a dominant A1 will often allow the surgeon to dissect the dome of an ACOM aneurysm without rupture. However, even after a temporary clip is placed on the proximal ICA, the fundus may receive significant inflow retrograde from a large PCOM artery. In addition, a temporary clip on the ICA will often significantly interfere with dissection and clipping of a PCOM aneurysm. Therefore, adenosine arrest provides a temporary reduction of inflow from all these vessels allowing the surgeon to dissect the neck of the aneurysm and apply a clip without rupture of the aneurysm.

Once the aneurysm is successfully clipped, the surgeon must once again evaluate the integrity of the PCOM artery, perforators and the anterior choroidal artery and make sure that there is no residual flow into the dome of the aneurysm. Because the neck of junctional PCOM aneurysms involves both the ICA and PCOM artery, complete occlusion of the aneurysm may be difficult and may require the use of multiple clips. Intraoperative use of microdopler and indocyanic green angiography are useful adjuncts especially when evaluating the patency of small perforating arteries. Although intra-operative angiography is not effective for evaluating the patency of small perforators, it is often effective in detecting iatrogenic occlusion or stenosis of larger vessels (such as PCOM artery) and residual flow into the dome of the aneurysm. In fact, Alexander *et al*, found that giant aneurysm and PCOM location were independent predictors of residual aneurysm (detected via intra-op angiography) requiring clip adjustment.[3]

Although clipping of PCOM aneurysms may occasionally be met with complications, outcomes tend to be good for the majority of the cases. Wirth *et al*, retrospectively reviewed operative morbidity amongst unruptured aneurysms.[58] He found that PCOM aneurysms had the lowest operative morbidity (5%) compared to aneurysms in other locations (MCA 8%, ICA 12%, ACOM 16%). Although data from this study is somewhat old, and techniques and microscopes have improved surgical outcomes, PCOM aneurysm in general continue to be among the least complicated aneurysms to treat surgically. However, because of the emergence of endovascular therapy and significant technical advancements made with stent/balloon-assisted embolization, aneurysms that come to surgical management will likely be larger and more complicated.

It is in the experience of the senior author (GWB) that the complexity of aneurysms undergoing surgical clipping has increased with the advent of endovascular treatment as all those with a small or moderate sized necks would be coiled.

### Coil Embolization of PCOM Aneurysms

Since the data published in international subarachnoid hemorrhage aneurysm trial (ISAT), endovascular treatment of intracranial aneurysms has become more common.[36] More recent technical advancements with the use of stent/balloon-assisted embolization have increased the number of aneurysms that are amenable to coil embolization. This is particularly true for PCOM aneurysm given the proximal location along a first order intracranial artery. Angiography views of the neck of the aneurysm and its parent vessels are more easily obtained for PCOM aneurysm compared to ACOM or MCA aneurysms. In addition, microcatheter access to these aneurysms is typically much simpler than paraclinoid ICA aneurysms. The fundus of PCOM aneurysm is typically in line with the communicating segment of the ICA whereas microcatheter access to paraclinoid aneurysm often requires a second turn immediately after the carotid siphon. For these reasons, coiling of PCOM aneurysms tends to relatively uncomplicated compared to aneurysms located in other parts of the anterior circulation. One can find evidence for this in a subgroup analysis of elderly patients (65 and older) enrolled in the ISAT trial.[47] Although no difference in functional outcome was found between endovascular and surgical patients overall, the proportion of patients with ICA aneurysms (including PCOM aneurysms) who were independent at one year was significantly greater in the endovascular cohort (72% *vs*. 52%) whereas the proportion of independent patients with MCA aneurysms was significantly greater in the surgical cohort (86.7% *vs*. 45.5%). Therefore, despite the significant challenges encountered with navigation in tortuous vessels found in the elderly, coiling of aneurysms along the ICA is generally associated with good outcome. This subgroup analysis does not differentiate between PCOM aneurysms and aneurysms found elsewhere along the ICA; however, given the difficulties encountered during endovascular treatment of clinoid aneurysms (particularly in a tortuous carotid siphon), endovascular treatment is likely associated with better outcomes than reported in this study.

The major drawback of endovascular embolization is the high recurrence rate. Raymond *et al*, reported an evidence of recurrence in 33.6% of patients on follow up and major recurrence requiring retreatment in 20.7% of patients.[44] Factors associated with recurrence included aneurysm size, treatment during acute phase of rupture, incomplete initial occlusion, and duration of follow-up. There was no statistically significant association between aneurysm location and recurrence in this study; however, PCOM aneurysm had the second highest recurrence rate (37.2%). In addition, a study looking at major recurrence of aneurysms treated during the ISAT trial found that coiled aneurysms in the PCOM location had a significantly higher risk of recurrence requiring re-embolization.[8] Despite this high risk of recurrence, the risk or rebleeding is relatively low varying from 0.6% to 2.6%.[2,8,44] The low risk of hemorrhage is likely related to close follow up of patients enrolled in this study and retreatment of recurrent. Of interest, Aikawa found that four out of the six hemorrhages in his retrospective review occurred in large PCOM aneurysm (15-26 mm, *P*<0.05) making these aneurysms significantly prone to recurrence and rehemorrhage.[2]

Songsaeng *et al*, recently found five morphological factors in coiled PCOM aneurysms, which were predictive of initial occlusion and long-term stability on follow-up.[49] Two of these factors (small size, and dome to neck ration < 2) are known and supported by other studies. Another factors described is a small size of PCOM artery. Because one cannot protect PCOM arteries with assistive devices such as balloons, stents or framing coils, it also makes sense that a smaller PCOM artery is associated with a higher initial occlusion rate and long-term stability of the aneurysm. The last two factors described in this study are an ICA-fundus angle of 160-180° and a posteroinferior dome orientation. Although one can argue that there is an increased stability of the microcatheter in the fundus of these aneurysms allowing for a tighter coil-pack, the contribution of flow dynamics and vessel stress is unknown. However, studies such as this may help improve the selection of patients for endovascular treatment.

The most important risk factor for rehemorrhage of a coiled aneurysm is an incomplete initial embolization. Johnston *et al*, demonstrates this correlation in a retrospective review of 1001 patients undergoing either clipping or coiling of a ruptured aneurysm.[23] Although the extent of aneurysm occlusion was based on a subjective description from the operative report, a strong correlation was found between rehemorrhage and residual aneurysm. Risk of rehemorrhage increased from 1.1% in completely occluded aneurysm to 17.6% in a partially treated aneurysm where residual filling of the dome was left untreated. Also, the median time to rerupture was only three days. This finding argues against partial coiling of a wide-neck aneurysm (mainly involving the dome of the fundus) followed by definitive stent assisted coiling at a later date. Although the rate of rehemorrhage in PCOM aneurysm was not analyzed separately, a trend toward an increased risk of rerupture was found in ICA aneurysms (*P* = 0.072). Reasons for this trend include an increased risk of recurrence, an increased risk of residual aneurysm and/or an increased risk of rupture from a residual aneurysm. This study also demonstrated a trend toward an increased risk of new disability with greater degree of initial occlusion achieved during the initial treatment indicating the importance of weighing the risk of complication during the procedure versus the risk of rehemorrhage.

PCOM aneurysms may be particularly difficult to coil in a ruptured setting without the assistance of a stent or balloon. Many of these aneurysm have an elongated fundus such that even though the dome to neck ratio appears favorable (based on the height of the aneurysm), a residual neck is often left unsecured. This is particularly true when the neck partially or totally involves the origin of the PCOM artery. Although stent-assisted embolization is very useful and sidewall aneurysms of the ICA and balloonassisted embolization is effective for embolization of bifurcation aneurysms, it remains extremely difficult to protect the PCOM artery when the neck of the aneurysm involves its origin. Zada *et al*, evaluated treatment of PCOM aneurysms with a fetal PCA origin.[60] Out of 30 patients, only 5 underwent coil embolization (*P* = 0.017). This bias toward surgical treatment of such aneurysms was confirmed by many of the commentators as well indicating that when a fetal type PCOM is found at the origin of the aneurysm, an optimal dome to neck ratio is necessary to effectively coil such aneurysms. This article also stresses the importance of formulating a treatment plan for each aneurysm on an individual basis and weighing the overall risks and benefits of each treatment without bias.

### Morbidity and Mortality of Coiling *vs*. Clipping

Unfortunately, with the exception of the ISAT elderly subgroup analysis, there is very little data that stratifies morbidity and mortality base on aneurysm location. The ISUIA probably provides the most accurate data for coiling and clipping of aneurysms.[56] The total prevalence of adverse outcomes at one year amongst the clipping cohort was 12.6% *vs*. 9.8% for the coiling cohort. There was no significant difference in mortality between the two groups (2.7% clipping *vs*. 3.4% coiling). The morbidity was slightly higher in the surgical group (9.9% clipping *vs*. 6.4% coiling), but not statistically significant. Of importance in this study is the measurement of cognitive impairment, which comprised nearly half the morbidity in each group. As mentioned earlier, the morbidity and mortality for PCOM aneurysm is likely much less given that aneurysms in this location are typically easier to treat than aneurysms in other locations. A more recent study on the morbidity and mortality of unruptured intracranial aneurysms found that coiling was associated with a lower mortality and morbidity (8.35% *vs*. 3.69%).[4] While this data was statistically significant in favor of coiling, the lack of randomization makes it difficult to generalize this data to all aneurysms. One must consider that the complexity of the aneurysms referred for surgical management has likely increased with time. This is consistent with cumulative outcome data showing an increased prevalence of adverse outcomes with surgical clipping following the ISAT trial.[31] This study also shows a decrease in the prevalence of adverse outcomes for endovascular coiling, which is likely related to the learning curve and technological improvements made in the field of endovascular surgery. Although the introduction of stent/balloon-assisted embolization has narrowed the gap between the complexity of aneurysms referred for surgical *vs*. endovascular treatment, many studies have shown that the risk of adverse outcome is higher when an assistive device is used.[20,53,55]

In addition to the immediate risks of morbidity and mortality associated with each procedure, one must also weigh the long-term risks including the risk of rebleeding and the risk of morbidity associated with each retreatment session. Mitchell *et al*, calculated the projected mortality rates for patient’s enrolled in the ISAT trial based on life expectancy and rates of rehemorrhage.[35] He found that the mortality benefit afforded by coiling might not hold true for patients less than 40 years of age. In addition, although long-term follow up of the patients enrolled in the ISAT trial showed a durable mortality benefit for the coil embolization cohort, the morbidity benefit was lost at five years.[37]

Because of the lack of bias and inclusion of morbidity associated with retreatment is missing from many of the published studies to date, one cannot take a dogmatic approach when choosing the treatment modality for aneurysms in which clinical equipoise exists (such as PCOM aneurysms). The decision regarding treatment is best made using a multidisciplinary approach. Gerlach *et al*, recently published a series of patients with unruptured intracranial aneurysms in which such an approach was used regarding treatment.[15] While the series is small (142 patients) and involves just one treatment center, the outcomes form both treatment modalities were favorable. No mortality was reported in either cohort. Neurological sequelae occurred in 6.3% of surgical patients and 7.7% of endovascular patients and a modified Rankin score < 2 was found in a very small proportion of each cohort (2.1% clipping *vs*. 2.7% coiling). This study stresses importance of weighing the challenges involved in treatment of each individual patient in order to reduce the risk of complications. The proportion of patients treated via each modality will likely vary by center depending on the experience of the surgeon and interventionalist.

### Occulomotor Nerve Palsy and PCOM Aneurysms

Occulomotor nerve palsy (OMNP) is a relatively common presenting sign of a PCOM aneurysm. Roughly 20% of PCOM aneurysms have OMNP on presentation and 80% of aneurysms with OMNP were located in the PCOM origin.[11,33] Roughly 70% of patients present with a complete OMNP, while 30% present with a partial OMNP. The sequence of impairment typically involves pupillary dilation followed by ptosis and then in extra-ocular paralysis/paresis. However, up to 17% of patients present with a pupil sparing OMNP[28] and recovery of OMNP in these patients is similar to other patients with a partial OMNP.[48] OMNP (without SAH) is usually secondary to some conformational change in to dome of the aneurysm and often indicates an impending rupture. However, the time from pupillary dilation to rupture varies from a few days to many months (average 29 days).[41,54]

Recovery of OMNP secondary to a PCOM aneurysm was initially observed after surgical clipping of the aneurysm. The main factor associated with recovery of OMNP was the time to treatment. Botterell *et al*, and Grayson *et al*, found that presence of OMNP greater than 10 days prior to operative intervention was associated with a reduced likelihood of a full recovery.[7,16] Leivo *et al*, performed a meta-analysis of a patient presenting with OMNP and a PCOM aneurysm, finding direct correlation between time to treatment and the likelihood of a full recovery.[33] 63% of patients operated on within two weeks of OMNP symptoms made a complete recovery, while only 30% of patients with symptoms greater than two weeks made a complete recovery and only 17% of patient with symptoms greater than one month made a complete recovery. Recovery of third nerve function was observed for up to one year. A major weakness of this study includes lack of standardized occulomotor testing and lack of a definition of complete recovery. Most authors consider lack of ptosis and double vision consistent with a complete recovery; however, recovery of pupillary function is variable and not directly correlated with recovery of double vision.[13] Kyriakides also evaluated factors related to complete recovery of OMNP.[30] Although he did not find any association between timing of operative intervention and recovery of OMNP, he did find an association between the degree of pre-operative deficit and the likelihood of recovery. Of interest, both Lievo and Kyriakides did not find any improvement in recovery with aspiration/decompression of the aneurysm. One can argue that manipulation of the third nerve during decompression subjects the nerve to further mechanical injury, which may or may not be reversible.

Because there is no correlation between surgical decompression of the third nerve and resolution of OMNP, one can also make an argument that the source of the OMNP is pulsation rather than direct decompression. Consistent with this hypothesis is the fact that coil embolization of PCOM aneurysms can also relieve OMNP without removal of direct mass effect. Birchall *et al*, described a series of three elderly patients with SAH and partial OMNP due to a PCOM aneurysm who recovered completely after coil embolization.[5] However, a larger case series by Stiebel-Kalish *et al*, had slightly less favorable results.[50] None of the 11 patients in this series had complete recovery of OMNP, although 10 patients did experience significant improvement with no double vision on primary gaze. One of the reasons for lack of improvement in these patients is that all patients had complete OMNP, which reduces the likelihood of a complete recovery. Hanse *et al*, also reviewed a series of 21 patients with OMNP who underwent coil embolization.[18] Out of 15 patients with complete OMNP, only three patients (20%) had complete recovery while five out of six patients (83%) with partial OMNP had complete recovery of third nerve function. Unlike the surgical series, neither of these series showed a correlation between time to treatment and recovery of third nerve function. Kassis *et al*, recently published a series of 20 patients with OMNP due to a PCOM aneurysm and did find a correlation between time to treatment and recovery of third nerve function.[27] 58% of patients treated within seven days made a complete recovery while none of the patients treated after seven days made a complete recovery (*P* = 0.016). In addition, Kassis *et al*, found that patients who presented with SAH had a higher likelihood of recovery than patients with OMNP and an unruptured aneurysm (*P* = 0.007). Based on this finding, one can hypothesize that the mechanism for OMNP in patients with SAH may include an inflammatory effect and/or microvascular effect secondary to the blood in the cisterns and that this effect may have a more favorable natural history and may be responsive to corticosteroids.

Two case series have been published comparing OMNP outcome between surgical and endovascular treatment of PCOM aneurysms. Chen *et al*, described a series of 13 patients of whom 6 were treated with coil embolization and 7 were treated with surgical clipping.[9] Only two patients in the endovascular cohort made a complete recovery, while six patients in the surgical cohort made a complete recovery (*P* = 0.05). Roughly 50% of patients in each cohort had partial OMNP, and these patients had a higher likelihood of complete recovery than patients with a complete OMNP (*P*=0.03). The second series comparing the two treatment modalities involves a retrospective review of 17 patients with unruptured PCOM aneurysms and OMNP.[1] Although an association was found between age, history of diabetes mellitus and time to treatment, there was no significant difference in recovery between the surgical and endovascular cohorts. Two factors may have negative outcomes in the surgical cohort in this series. The percentage of patients with complete OMNP was higher in surgical cohort than in the endovascular cohort (14% *vs* 30%). In addition, all surgeries involved opening of the aneurysm dome and direct decompression of the third nerve, which may have had a negative impact on its recovery. Unfortunately, without a randomized blinded study, one cannot make a concrete conclusion about the efficacy of one treatment over the other; however, both treatment modalities appear to offer a reasonable chance of recovery. However, because cohorts in the article by Chen *et al*, are more comparable than the cohorts in article by Ahn, one can make an argument that patients with OMNP may have a higher likelihood of recovery with clipping than with coiling; however, because these studies are small and non-randomized, one cannot conclude that clipping provides a concrete benefit in terms of recovery of third nerve function.

## SUMMARY

PCOM aneurysms are one of the most frequently encountered aneurysms. Unique anatomical variations in this aneurysm include the size of the PCOM artery and variable involvement of this artery in the neck of the aneurysm. Anterior clinoidectomy, temporary clipping, adenosine induced cardiac arrest, and intraoperative angiography are useful adjuncts during surgical clipping of these aneurysms. Coil embolization is also an effective treatment alternative particularly in the elderly population. However, coiled PCOM aneurysms have a particularly high risk of recurrence and must be followed closely. PCOM aneurysms with an elongated fundus, true PCOM aneurysms, and aneurysms associated with a fetal PCOM artery may have better outcomes with surgical clipping in terms of completeness of occlusion and preservation of the PCOM artery. However, as endovascular technology improves, endovascular treatment of PCOM aneurysms may become equivalent or preferable in the near future.

One in five patients with a PCOM aneurysm present with OMNP with or without SAH. Factors associated with a higher likelihood of recovery include time to treatment, partial third nerve deficit, and presence of SAH. Both surgical and endovascular therapy offer a reasonable chance of recovery. Based on level 2 evidence, clipping appears to offer a higher chance of OMNP recovery; however, coiling will remain as an option particularly in elderly patients or patients with significant comorbidity.
